# Sequestration of Exogenous Volatiles by Plant Cuticular Waxes as a Mechanism of Passive Associational Resistance: A Proof of Concept

**DOI:** 10.3389/fpls.2020.00121

**Published:** 2020-02-25

**Authors:** Xicotencatl Camacho-Coronel, Jorge Molina-Torres, Martin Heil

**Affiliations:** ^1^ Laboratorio de Ecología de Plantas, CINVESTAV-Irapuato, Departamento de Ingeniería Genética, Irapuato, México; ^2^ Laboratorio de Fitobioquímica, CINVESTAV-Irapuato, Departamento de Biotecnología y Bioquímica, Irapuato, México

**Keywords:** antifungal volatiles, antracnosis, cuticle, defense, pathogen, plant disease

## Abstract

Numerous plant-derived volatile organic compounds (VOCs) induce the expression of resistance-related genes and thereby cause an “associational resistance” in neighbouring plants. However, VOCs can also be sequestered by plant cuticular waxes. In case that they maintain their biological activity, such sequestered VOCs could generate a “passive” associational resistance that is independent of any gene expression in the receiver. As a proof of concept, we used major components of the cuticular wax layers of the tree, *Parkinsonia praecox*, and conidia of *Colletotrichum lindemuthianum,* a fungal pathogen that has not been reported to infect *P. praecox*. Wax layers were re-constituted on glass slides and exposed to each of 20 pure VOCs for 1 d and then to ambient air for 1 d or 15 d. Gas chromatography-mass spectrometry (GC-MS) analyses showed that all 20 VOCs were sequestered by the re-constituted wax layers. Exposure to 18 of the VOCs significantly inhibited the germination of *C. lindemuthianum* conidia on these wax layers after 1 day of exposure to ambient air. Four of the VOCs: 4*Z*-heptenol, farnesene, limonene, and 2*E*-decenal, inhibited germination rates to less than 25% of the controls. After 15 d, all VOCs were still detectable, although at strongly reduced concentrations, and no significant inhibition of conidial germination could be detected anymore. Exogenous VOCs can be sequestered by the components of plant cuticular waxes and maintain their biological activity, at least over a certain time span: an effect that could generate a transient “passive associational resistance” to pathogens.

## Introduction

Plants respond to biotic and abiotic stress with the release of a diverse array of VOCs. These VOCs can contribute to the resistance to herbivores and pathogens in the emitting plant itself (Heil, 2014; Ameye et al., 2018), but VOCs are also being discussed to explain the phenomenon of “associational resistance”: a resistance effect that is mediated by neighbouring plants (Himanen et al., 2010; Himanen et al., 2015). Associational resistance to herbivores is a frequently described phenomenon and might represent a mechanism that contributes to the beneficial effects of polyculture systems. Therefore, VOCs receive increasing interest as a potential tool in biological control (Himanen et al., 2015; Stenberg et al., 2015; Brilli et al., 2019; Mofikoya et al., 2019).

Two commonly discussed mechanisms to explain VOC-mediated associational resistance are the induction of resistance-related traits in the receiver plant (Heil, 2014; Castelyn et al., 2015; Kim, 2017; Ameye et al., 2018; Effah et al., 2019) and the sequestering of exogenous VOCs in plants and their subsequent release into the atmosphere (Himanen et al., 2010; Li and Blande, 2015; Mofikoya et al., 2017). For example, birch (*Betula* spp.) leaves gained associational resistance *via* the sequestering and re-release of herbivore-repellent VOCs that had been emitted from neighbouring *Rhododendron tomentosum* plants (Himanen et al., 2010; Himanen et al., 2015), and exogenous VOCs from neighbouring plants affected the preference of the specialist herbivore, *Plutella xylostella* for its host plant, *Brassica oleracea* (Li and Blande, 2015). Thus, Himanen & col suggested that exogenous VOCs can generate a “passive associational resistance” (Himanen et al., 2010; Himanen et al., 2015; Mofikoya et al., 2017). Nevertheless, concerns remain because under ambient conditions, VOCs are quickly diluted, dispersed, oxidised, or degraded—particularly under the influence of wind, ozone, and UV radiation—and thus, VOCs are unlikely to maintain in stable concentrations in the atmosphere (Holopainen and Blande, 2013; Niinemets et al., 2014; Li et al., 2016; Giron-Calva et al., 2017; Holopainen et al., 2017; Mofikoya et al., 2017).

However, the outer surface of the above-ground part of the plants represents a major battleground for plant-microbe interactions (Junker and Tholl, 2013). These surfaces are covered by a matrix collectively designated as (epi)cuticular waxes (Buschhaus and Jetter, 2011): complex mixtures of hydrophobic compounds such as long-chain esters—compounds chemically considered as waxes (Bruice, 2006)—and other lipophilic compounds such as saturated aliphatic hydrocarbon chains of at least 20 carbons, pentacyclic triterpenoids, and phenylpropanoids (Vogg et al., 2004; Kunst and Samuels, 2009; Buschhaus and Jetter, 2011; Hama et al., 2019). Thus, due to the lipophilic nature of these epicuticular waxes, it has been proposed that endogenous VOCs can accumulate in the epicuticular wax layers of plants (Widhalm et al., 2015). However, the same argument applies also to exogenous VOCs, i.e., VOCs that have been emitted from other parts of the plant or from neighbouring plants In fact, endo- and exogenous terpenes were reported from the epicuticular wax matrix of Scots pine (*Pinus sylvestris*) trees growing in their natural habitat (Joensuu et al., 2016). Many VOCs have antimicrobial effects, at least *in vitro*. For example, VOCs emitted from pathogen-challenged bean plants inhibited the germination of fungal conidia on leaf surfaces or *in vitro* (Quintana-Rodriguez et al., 2015; Quintana-Rodriguez et al., 2018b) and VOCs emitted from grapevine cultivars resistant to downy mildew reduced the infection rates of susceptible cultivars and had direct antifungal effects when tested *in vitro* or using leaf discs (Lazazzara et al., 2018).

Based on the above-mentioned anecdotic evidence showing i) that birch leaves sequestered VOCs from neighbouring *Rhododendron tomentosum* plants (Himanen et al., 2010) and ii) that plant-derived VOCs directly inhibited the germination of conidia of plant pathogenic fungi such as *Botrytis cinerea, Colletotrichum lindemuthianum, Fusarium oxysporum,* (Quintana-Rodriguez et al., 2015; Quintana-Rodriguez et al., 2018b) or *Plasmopara viticola* (Lazazzara et al., 2018), we hypothesized that exogenous VOCs can be sequestered by the cuticular waxes of a receiver plant without losing their antifungal properties and that this mechanism might contribute significantly to a “passive associational resistance” against pathogens, which is independent of any resistance gene expression in the receiver plants. The goal of the present study was to provide a proof of concept for this hypothesis.

**Figure 1:**
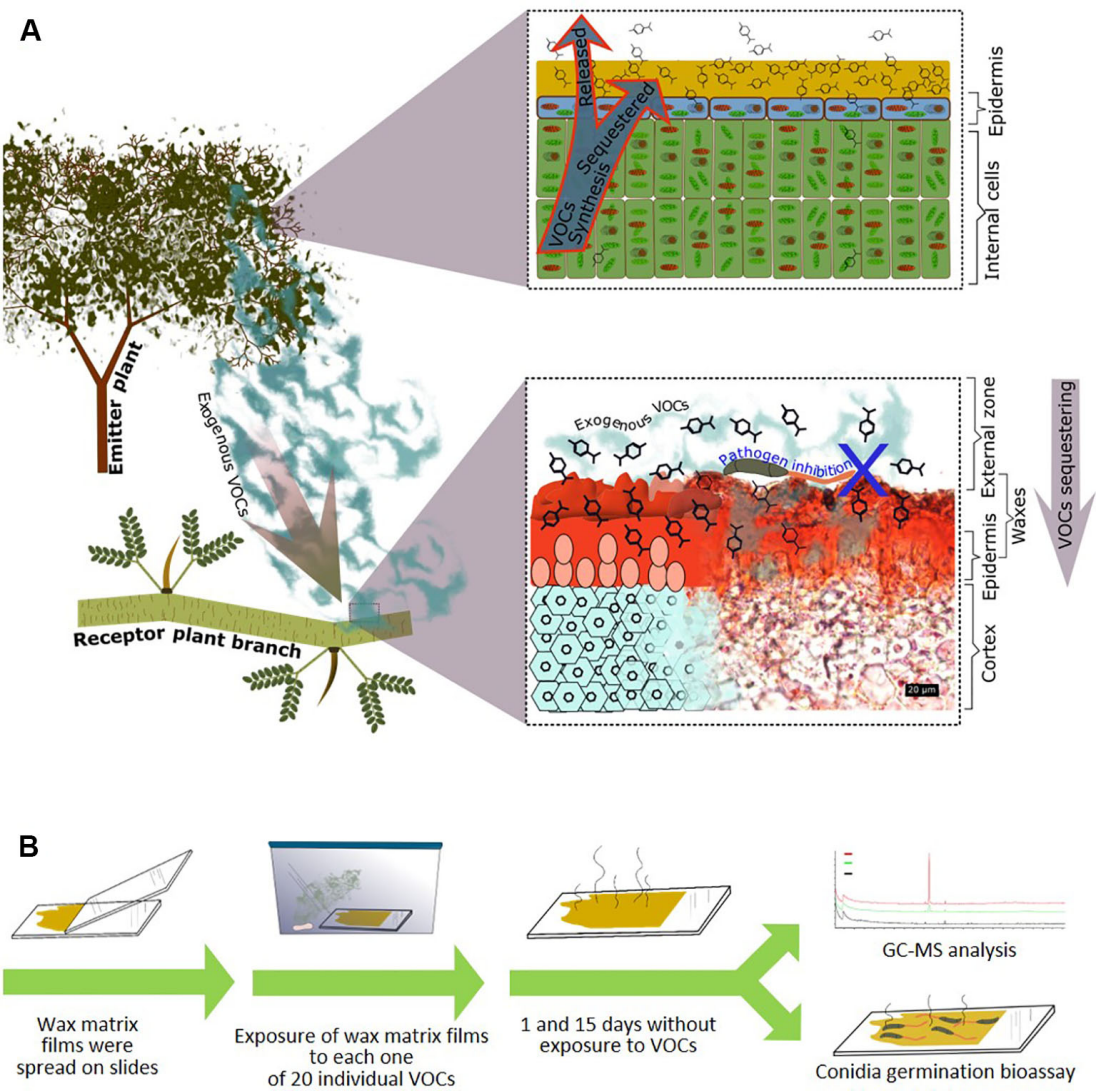
Passive associational resistance: Hypothesis and experimental approach. **(A)** We propose that—in addition to the endogenous volatile organic compounds (VOCs) that are retained in the cuticular waxes during the emission process (upper panel right side)—also exogenous VOCs can be sequestered by the cuticular waxes of a plant (here: *Parkinsonia praecox*) and maintain their antimicrobial effects. Lower panel right side: transversal section of a *P. praecox* branch, waxes stained red with Sudan III (photograph donated by Silvia Aguilar Rodríguez). **(B)** Experimental design: Wax matrix layers were re-constituted on glass slides and then exposed to each out of twenty individual VOCs at a theoretical concentration of 7.142 µM in a closed atmosphere. After subsequent exposure to ambient air, the wax layers were randomly assigned to gas chromatography-mass spectrometry (GC-MS) analysis or biotests (germination of conidia of *Colletotrichum lindemuthianum*).

To focus on the direct antifungal effects of VOCs-containing waxes and exclude any influences from a receiver plant, we employed an *in vitro* experimental setup (**Figure 1**). As a source of major compounds of natural cuticular waxes, we selected the native Mexican plant, *Parkinsonia praecox* (Ruiz & Pavon) Hawkins (Fabaceae), which is known for its thick layer of cuticular waxes (Aguilar Rodríguez et al., 2016; Aguilar Rodríguez et al., 2019). As a model pathogen, we chose *Colletotrichum lindemuthianum*. This fungal pathogen commonly infects bean and other plants within the Fabaceae and has been shown to be sensitive to exogenous VOCs during the initial colonisation of host plants (i.e., during spore germination of the leaf surface, see Quintana-Rodriguez et al., 2015; Quintana-Rodriguez et al., 2018b; Martins et al., 2019). However, *C. lindemuthianum* is not known to infect *P. praecox*. Hence, it appeared to be unlikely that the cuticular waxes of *P. praecox* contained compounds that have evolved specifically as a defence against this particular pathogen. Layers of cuticular wax compounds were re-constituted on glass slides and exposed to each out of a total of 20 pure VOCs. These VOCs were selected because they have been reported to be emitted from plants (Aggarwal et al., 2002; Huang et al., 2012; Liu et al., 2012; Dudareva et al., 2013; Tao et al., 2014; ul Hassan et al., 2015) and to have antimicrobial activity (Aggarwal et al., 2002; Huang et al., 2012; Liu et al., 2012; Tao et al., 2014; Quintana-Rodriguez et al., 2015; Quintana-Rodriguez et al., 2018b). After subsequent exposure of these wax layers to ambient air for 1 or 15 d, we used gas chromatography-mass spectrometry (GC-MS) to quantify VOCs in the wax layers and spore germination assays to test for antifungal effects of the VOC-containing wax layers. Due to the absence of any plant, or leaf, from the assay system, any inhibitory effect on spore germination observed after 1 or 15 d could be clearly linked to VOCs that had been sequestered by the compounds in the re-constituted wax layers and remained biologically active over the considered time span.

## Materials and Methods

Individual branches of plants of *Parkinsonia praecox* (Ruiz & Pavon) Hawkins (Fabaceae) were collected at a natural site in Tlacotepec de Benito Juárez, Puebla, México (18° 36´ 38.4′′ N, 97° 42′ 06.1′′ W). The plant species was identified by Silvia Aguilar Rodríguez (Facultad de Estudios Superiores Iztacala, UNAM, México) according to (Burkart and Carter, 1976). The strain 1088 of *Colletotrichum lindemuthianum* (Sacc. & Magn.) Briosi & Cav. had been isolated in the state of Durango in México from climbing-type cultivars of bean (González et al., 1998; Luna-Martínez et al., 2006) and was donated by June Kilpatrick Simpson Williamson (CINVESTAV-Irapuato, México). The volatiles 1-octen-3-ol; 2*E*,4*E*-nonadienal; 2*E*-hexenyl acetate; 5-hexenyl acetate; 4*Z*-heptenol; β-caryophyllene; decanal; eugenol; farnesene; heptanal; limonene; linalool; methyl jasmonate; methyl salicylate; ocimene; octanal; terpineol; 2*E*-decenal; 2*E*-dodecenal; and 2*E*-tridecenal, were purchased at analytical grade from Sigma-Aldrich, St. Louis, Missouri, USA.

Branches of *P. praecox* designated to histochemical analyses (photo in **Figure 1A**) were fixed in FAA solution (formaldehyde:glacial acetic acid:distilled water:ethanol, 10:5:35:50 v/v) until usage. Transverse sections with 15 µm of thicknesses were cut with a rotary microtome and then the sections were stained with Sudan III (Sigma-Aldrich, St. Louis, Missouri, USA), and observed with Leica DM750 microscope (Leica microsystems, Wetzlar, Germany) to detect lipidic compounds.

For the VOC-sequestration analyses, cuticular waxes were removed mechanically from fresh branches by gently scraping the surfaces of the *P. praecox* branches with a scalpel, avoiding damage to the epidermis, weighed and suspended in chloroform (Sigma-Aldrich, St. Louis, Missouri, USA) at 60°C to a final concentration of 0.1 g ml^-1^ and then filtered through Whatman^®^ qualitative filter paper, Grade 4. The solvent was evaporated at room temperature to obtain purified wax compounds. To analyse the major compounds in the re-constituted wax layers, 0.15 mg of waxes were dissolved in 1 ml of chloroform containing as an internal standard tetracosane at a final concentration of 1µg µl^-1^. 500 µl of that solution was placed in a 2 ml Eppendorf^®^ tube, the chloroform was evaporated with N_2_ flux. For derivatization, we added 100 µl of bis-N,N-(trimethylsilyl)trifluoroacetamide (BSTFA, Sigma-Aldrich, St. Louis, Missouri, USA) and 100 µl of pyridine and incubated 30 min at 70°C. The samples were dried and re-dissolved in 200 µl chloroform. Analysis was performed using an Agilent 7890 series gas chromatograph interfaced to an Agilent 5975 electronic ionization mass-selective triple axis detector (Agilent Technologies, Santa Clara CA, USA), using the following conditions: HP-5MS column (Agilent 15 X 0.25, 0.25, Agilent Technologies, Palo Alto, CA, USA), injection volume 1µl, injection port temperature 270°C, oven program: initial temperature 70°C, hold 2 min, increased at 20°C min^-1^ to 200°C, hold 2 min, then increased at 3°C min^-1^ to 310°C and hold 30 min. Compounds were preliminarily annotated using NIST MS Search Program v.2.0g, Library version 11 Mass Spectral DataBase, and AMDIS deconvolution software version 2.71 (National Institute of Standards and Technology. US Department of Commerce).

To generate the re-constituted wax layers for VOC sequestration assays, 100 mg waxes were re-suspended in 1 ml of chloroform, and 80 µl of this suspension (containing 8 mg of waxes) was spread over an area ca 6 cm^2^ on a glass slide. After solvent evaporation, the slides were introduced individually into 350 cm^3^ Magenta™ plant culture boxes (Bioworld, Ohio, USA). The VOCs were dissolved individually in chloroform at 0.05 M and 50 µl of this solution was pipetted on a Whatman filter paper disc of 0.5 cm located in the bottom of the Magenta culture boxes without direct contact with the wax matrix layers. This concentration was chosen based on earlier work (Quintana-Rodriguez et al., 2015). Assuming complete evaporation and no loss or surface adsorption of the VOC, this setup generated a concentration of 7.142 µM VOC in the atmosphere of Magenta™ boxes, which is within the range of VOCs as found in the headspace of resistance-expressing bean plants (Quintana-Rodriguez et al., 2015). Subsequently, the boxes were closed for 24 h. After 24 h, the slides with the wax layers were removed and exposed to ambient air in the laboratory for 1 or 15 d, to then be assigned randomly to either chemical analyses (n=3 technical replicates per VOC) or biotests (n=7 biological replicates per VOC).

To quantify the sequestered VOCs, the wax layers were mechanically removed from the glass slides and re-suspended in 200 µL of chloroform. Direct analysis was performed using an Agilent 7890 series gas chromatograph interfaced to an Agilent 5975 electronic ionization mass-selective triple axis detector (Agilent Technologies, Santa Clara CA, USA), using the following conditions: HP-5MS column (Agilent 15 X 0.25, 0.25, Agilent Technologies, Palo Alto, CA, USA), injection volume 1 µl, injection port temperature 180°C, oven program: initial temperature 60°C, increased at 5°C min^-1^ to 80°C then increased at 8°C min^-1^ to 210°C as final temperature. VOCs were identified by comparison of retention times and mass spectra with those of the standard compounds and quantified based on the peak areas, using calibration curves that had been generated using pure compounds at 5 different molar concentrations: 0.005, 0.05, 0.1, 0.25, and 0.5 mM in chloroform.

To test for the biological activity of the sequestered VOCs, the wax layers on the glass slide were inoculated with conidia of *C. lindemuthianum*. As controls, we used wax layers that had been subjected to the same procedure as described above but exposed to ambient air instead of a VOC headspace. To collect the conidia for the biotests, we poured 2 ml of tween at 0.05% in distilled water over mycelia of *C. lindemuthianum* that had been cultivated over 15 d on PDA medium. The resulting suspension was adjusted with distilled water to a concentration of 1 X 10^7^ conidia ml^-1^ (Quintana-Rodriguez et al., 2015), quantified by counting using a hemacytometer (Hausser Scientific, Horsham, Pennsylvania, USA) and a Leica DM750 microscope (Leica microsystems, Wetzlar, Germany) at 40X. The surfaces of the VOC-exposed waxes matrix were inoculated with 50 µl of this suspension and introduced into a Petri dish with 2 ml of distilled water to maintain the humidity. After 48 hours, the slides were stained with lactophenol blue (Sigma-Aldrich, St. Louis, Missouri, USA) to count the germinated and non-germinated spores using the microscope.

## Results

**Figure 2:**
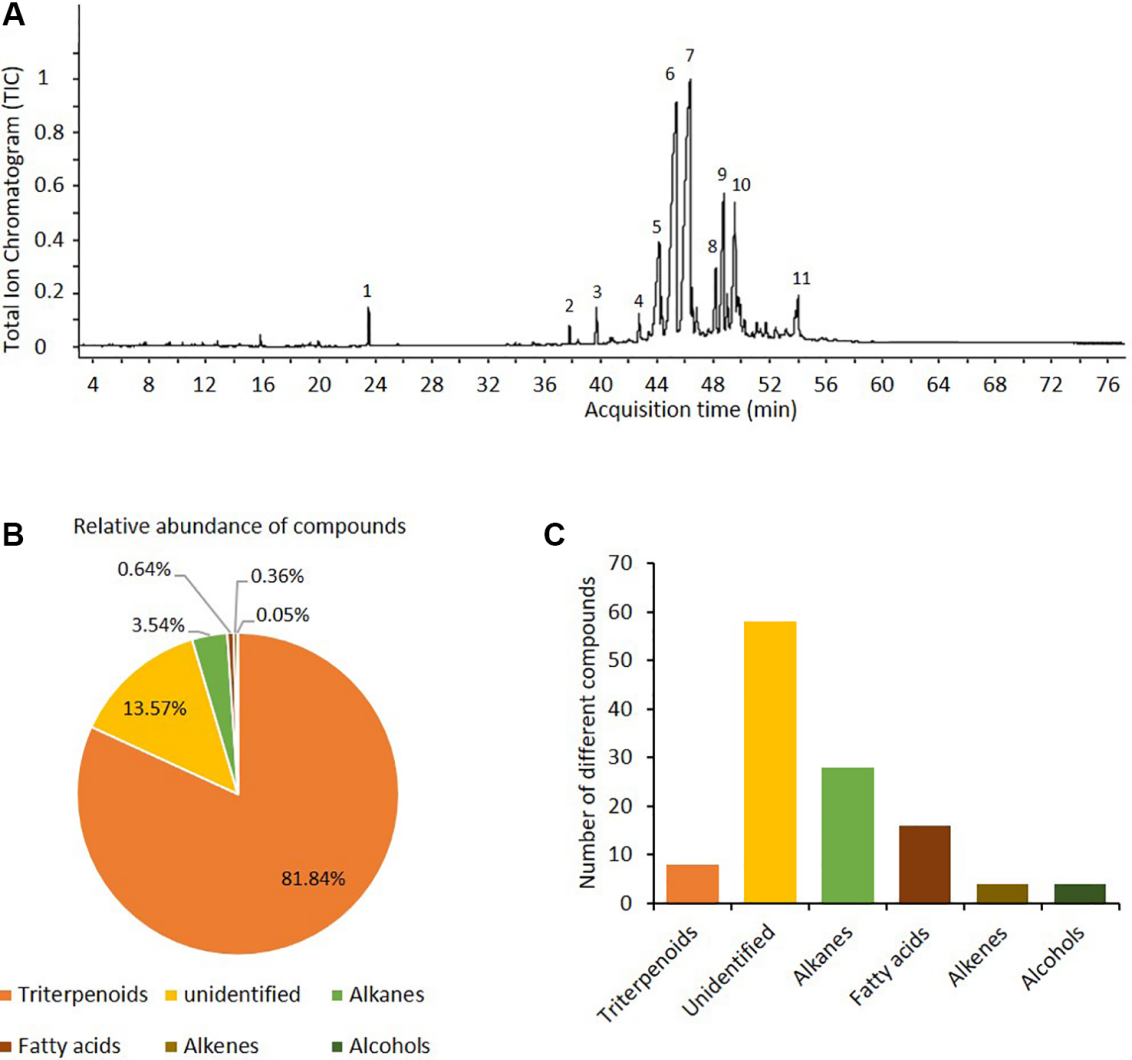
The epicuticular waxes matrix of *Parkinsonia praecox* contained mainly triterpenoids. **(A)** Representative chromatogram of compounds from cuticular waxes of *P. praecox.* Preliminary identification of compounds (for retention times and mass spectra, see **Figure A**, **Supplementary Material**): **1**) tetracosane (internal standard); **2**) tocopherol (triterpenoid 1); **3**) nonacosane (alkane 1); **4**) germanicol (triterpenoid 2); **5**), triacontane (alkane 2); **6**) hopenone b (triterpenoid 3); **7**) lupenone (triterpenoid 4); **8**), cyclolanostanol-derivative 1 (triterpenoid 5), **9**) oxolanostadienoate-derivative (triterpenoid 6); **10**) betulin (triterpenoid 7); and **11**) 24-methylene-cycloartenol (triterpenoid 8). **(B)** Relative abundance of the dominant compound classes in wax matrix. **(C)** Numbers of different compounds (i.e., distinct peaks in the GC-spur) that were assigned to each class of compounds found in the wax matrix (see **Figure B**, **Supplementary Material**).

Ten compounds were quantitatively dominant in the chloroform-based preparations of the cuticular waxes of *P. praecox* (**Figure 2**). Among these ten dominant peaks (**Figure 2A**), seven compounds were preliminarily identified as triterpenoids (betulin, germanicol, hopenone, lupenone, tocopherol, and derivates of cyclolanostanol, cycloartenol and of oxolanostadienoate), two as alkanes (nonacosane and triacontane), and one as a tocopherol-derivate (**Figure 2A**, **Figure A** in the **Supplementary Material**). Quantitatively, terpenoids compounds made up ca. 85% of the chloroform-soluble wax matrix (**Figure 2B**). In total, the preliminary annotation identified > 50 compounds; among these, nine were triterpenoids, 28 alkanes, 16 fatty acids, four alkenes and four alcohols (**Figure 2C**, **Figure B** in **Supplementary Material**).

**Figure 3:**
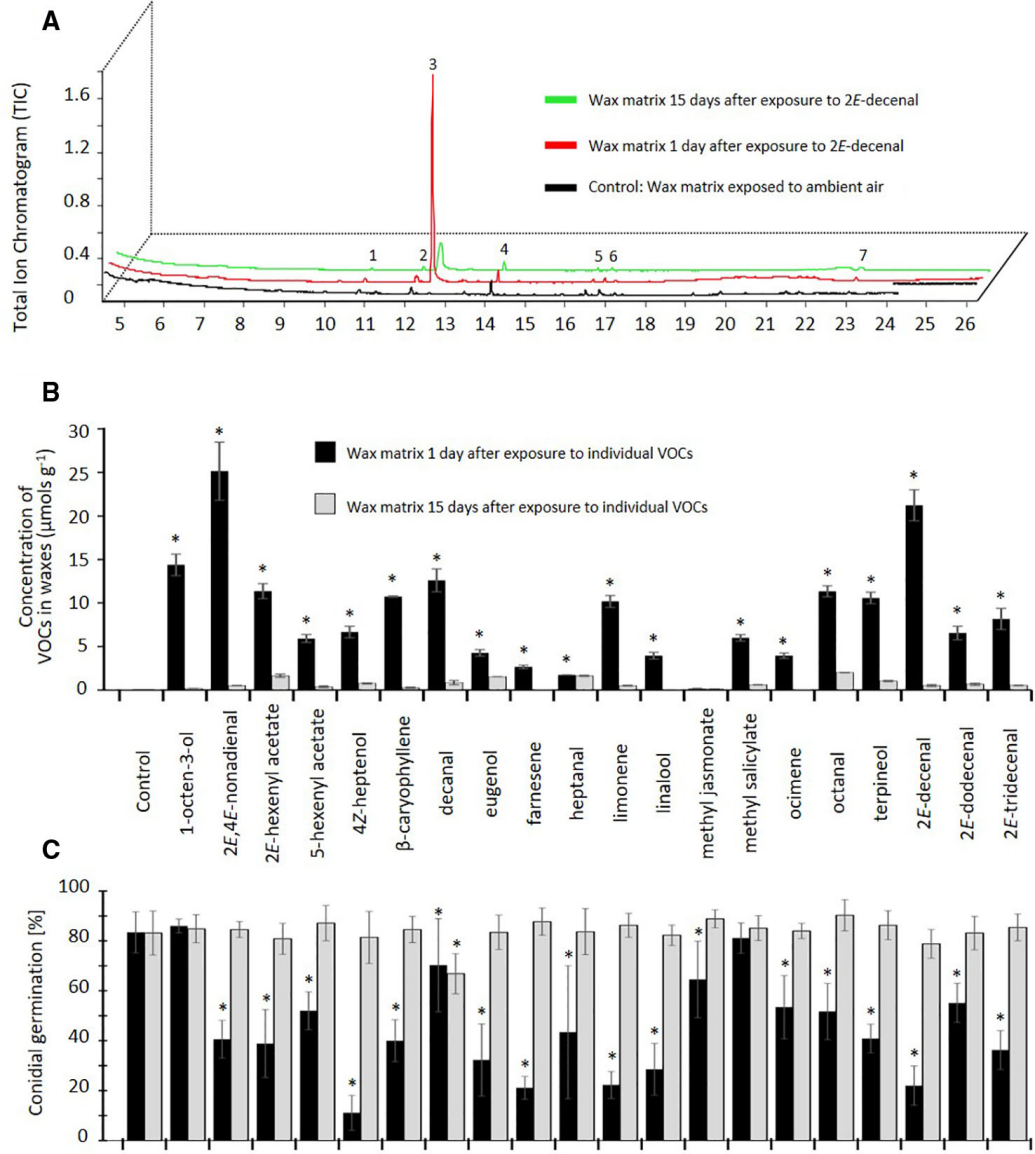
Proof of concept: Volatile organic compounds (VOCs) can be sequestered by epicuticular waxes and maintain their antifungal effect. **(A)** Chromatograms of epicuticular wax matrices of *Parkinsonia praecox* exposed to 2*Z*-decenal and subsequently to ambient air for 1 day (red) or 15 d (green) or exposed to ambient air only (control: black). Peak numbers: **1**,**2**) not identified; **3**) 2*Z*-decenal; **4**) 4-undecanol; **5**) 2,6,10,15-tetramethyl-heptadecane; **6**) 2,4-bis(1,1-dimethylethyl)-phenol; **7**) heneicosane. **(B)** The concentration of VOCs in wax matrices after exposure to at a theoretical concentration of 7.142 µM in a closed atmosphere and subsequent exposure to ambient air for 1 day (black bars) or 15 d (grey bars). Control: wax matrices exposed to ambient air only. Asterisks indicate significant differences between the concentration of sequestered VOC detected at day 1 and day 15 (p < 0.05, n=3 technical replicates, t-test. **(C)** Germination rate of *Colletotrichum lindemuthianum* conidia on VOC-exposed wax matrices. Asterisks mark germination rates significantly lower than controls (p < 0.05, Dunnett test, n=7 biological replicates). Bars in **(B, C)** indicate means, error bars indicate ± 1 SD.

All 20 tested VOCs were sequestered by the re-constituted wax layers and could be detected at quantities of 0.2–25 µmol g^-1^ wax matrix, after 1 d of exposure to ambient air, whereas no other VOCs could be detected apart from minor impurities that also appeared in the control wax layers that only had been exposed to ambient air (**Figures 3A, B**). Fifteen of the VOCs were detected at concentrations > 4 µmol g^-1^, the highest concentrations observed being ca. 25 µmol g^-1^ for 2*E*,4*E*-nonadienal and 21 µmol g^-1^ for 2*E*-decenal. Only farnesene, heptanal, linalool, methyl jasmonate, and ocimene were detected at < 4 µmol g^-1^. All compounds could still be detected after 15 d exposure to ambient air, although at significantly lower concentrations as compared to day 1 (for all VOCs: p < 0.05, t-test, n = 3 technical replicates, **Figure 3B**).

Exposure of the wax layers to 18 out of the 20 VOCs significantly inhibited the subsequent germination of *C. lindemuthianum* spores, at least when the re-constituted wax layers were inoculated after 1 d of exposure to ambient air (p < 0.05, ANOVA and Dunnett test, n =7, **Figure 3C**). The strongest inhibition—with germination rates < 50% of the controls—was observed for 2*E*,4*E*-nonadienal, 2*E*-hexenyl acetate, 4*Z*-heptenol, β-caryophyllene, eugenol, farnesene, limonene, linalool, terpineol, 2*E*-decenal, and 2*E*-tridecenal. Lower, but still significant, inhibition effects (germination rates 50% -75% of controls) were observed for 5-hexenyl acetate, heptanal, ocimene, octanal, and 2*E*-dodecenal, whereas no significant effect could be observed for 1-octen-3-ol and methyl salicylate. However, this inhibitory effect was lost after 15 d of exposure to ambient air, with the only exception of decanal, which still caused a significant reduction in the proportion of germinated spores (p < 0.05, Dunnett test, n=7 biological replicates, **Figure 3C**).

## Discussion

Associational resistance to herbivores is a frequently described phenomenon. Several studies demonstrated that the sequestration of exogenous VOCs by leaves and their subsequent release into the plant´s headspace can contribute to this phenomenon (Himanen et al., 2010; Li and Blande, 2015; Mofikoya et al., 2017). Here, we demonstrate the potential role of cuticular waxes in a passive associational resistance to pathogens that is mediated by exogenous antimicrobial VOCs. We used an *in vitro* model system consisting of wax layers that had been re-constituted from chloroform-based extracts of the cuticular waxes of *P. praecox*. The detailed analysis of these waxes was beyond the scope of the present work. However, preliminary annotation identified the quantitatively dominating compounds as long-chain alkanes and alkenes and as triterpenoids. Long-chained lipophilic compounds, and even triterpenoids such as betulin, germanicol, and tocopherols, are typically reported from the (epi)cuticular waxes of plants (Jetter and Riederer, 1995; Jetter et al., 2006; Racovita and Jetter, 2016; Guo et al., 2017), including from species in the same plant family as *P. praecox*, the Fabaceae (Kim et al., 2007; Regasini et al., 2009; Buschhaus and Jetter, 2011). Long-chained alkanes and alkenes, but also triterpenoids, are typical components of the cuticular wax layers of species from semi-arid climates (Racovita et al., 2015), and triterpenoids even accounted for almost 65% by mass of the cuticular waxes on heather (*Calluna* spec.) leaves (Szakiel et al., 2013). The high proportion of triterpenoids indicates that our wax preparations contained components that are more representative of intracuticular waxes (van Maarseveen and Jetter, 2009). However, in general terms, the detected major compounds are frequently reported from plant cuticular wax layers. Thus, we conclude that our re-constituted wax-layers represent a suitable *in-vitro* model to study whether VOCs could be sequestered by compounds as they are typically found in plant cuticular waxes and maintain their antifungal properties under these conditions.

Indeed, the re-constituted wax-layers sequestered all of the 20 VOCs tested (1-octen-3-ol; 2*E*,4*E*-nonadienal; 2*E*-hexenyl acetate; 5-hexenyl acetate; 4*Z*-heptenol; β-caryophyllene; decanal; eugenol; farnesene; heptanal; limonene; linalool; methyl jasmonate; methyl salicylate; ocimene; octanal; terpineol; 2*E*-decenal; 2*E*-dodecenal; and 2*E*-tridecenal), and these VOCs were still detectable in the wax matrix after 15 d exposure to ambient air. Moreover, 18 out of 20 VOCs significantly reduced the germination rates of *C. lindemuthianum* conidia on these wax layers, at least after 1 d exposure to ambient air. This inhibitory effect was lost after 15 d, most likely due to the low concentrations of the remaining sequestered VOC within the wax matrix. Nevertheless, our observations demonstrate that VOCs maintain their antimicrobial activity even when they were sequestered by plant cuticular waxes.

The capacity of this wax matrix to sequester VOCs strongly differed among compounds and the amount of sequestered VOC did not correlate closely with the observed inhibitory effect on the germination of fungal conidia. For example, 2*E*,4*E*-nonadienal and 2*E*-decenal were detected at highest concentrations in the waxes, whereas lowest germination rates were observed on wax layers that had been exposed to 4*Z*-heptenol, eugenol, farnesene, limonene, linalool, or 2*E*-decenal. By contrast, only traces were detected for methyl jasmonate, but the spore germination on these wax matrices was slightly, but still significantly, inhibited. Finally, a relatively high concentration of decanal after 1 d was associated with a low inhibition effect, but this effect remained stable even after an almost complete loss of decanal after 15 d. These observations show that further factors need to be considered if we aim to fully understand the anti-fungal activity of plant waxes that had been exposed to exogenous VOCs.

Beyond doubt, the experimental conditions in this study were highly artificial, and several factors should be considered for an adequate interpretation of our observations. First, epicuticular waxes form complex crystal structures (Hama et al., 2019), in particular tubular crystal-like structures that consist mainly of nonacosan-derivatives. These tubular structures easily re-build from wax extracts (Jetter and Riederer, 1995), and nonacosane and triacontane were major compounds in the *P. praecox* wax preparations (**Figure 2**). Nevertheless, it appears to be unlikely that our re-constituted way layers had maintained a significant part of the natural, three-dimensional structure of *P. praecox* waxes. Therefore, our results indicate that the detailed three-dimensional structures of cuticular waxes are not likely to represent a crucial element for the sequestration of exogenous VOCs. Rather, the localisation of the VOC within the waxes and chemical reactions of the VOCs with other molecules are factors that might explain the low correlation between the amounts of different VOCs and the antimicrobial effects that we observe in our results. For example, VOCs rapidly become oxidised, and the oxidised molecules have much longer lifetimes than the original molecules (Holopainen et al., 2017) yet, they might have completely different effects on fungal pathogens.

Second, several components of plant epicuticular waxes have antimicrobial effects as well (Tholl, 2015), and the waxes used here were obtained from plants in their natural habitat and, thus, might still have contained ambient VOCs from the field site. Both factors represent potential sources of effects that might have influenced our observations. However, the waxes were obtained from the branches and stems of the plant, long-chained alkanes and triterpenoids are usually considered as transpiration barriers (Oliveira et al., 2003; Buschhaus and Jetter, 2011; Sharma et al., 2018), and to the best of our knowledge, *C. lindemuthianum* is not a natural pathogen of *P. praecox*. Altogether, these factors make it unlikely that the waxes of *P. praecox* contained any compounds that have specifically co-evolved to resist infection by *C. lindemuthianum*. Moreover, the experimental procedure consisted of two steps of dissolving the waxes matrix and subsequent evaporation, before subjecting the waxes matrix to the bioassays, and as controls we used wax matrix layers that had been subjected to the same procedure but then exposed to ambient air instead of a VOC. Although the appearance of trace amounts of exogenous VOCs in the controls indicates that the ambient air, as expected, was not completely clean, the inhibitory effects observed on the VOC-exposed wax layers were quite strong and statistically robust. Therefore, we conclude that germination rates significantly below those on the control waxes can most likely be attributed to the sequestered VOCs.

Finally, only one concentration of each VOC was used and the concentration of sequestered VOC in the wax layers was only quantified at two time points. Different VOCs strongly differ in their antimicrobial activity on a specific strain. For example, β-caryophyllene, eugenol, limonene, linalool, and terpineol are frequently reported to inhibit diverse bacterial and fungal strains (Aggarwal et al., 2002; Krist et al., 2007; Krist et al., 2008; Zhou et al., 2014; Pieri et al., 2016; Yoo and Jwa, 2018). Linalool, eugenol, 2*E*-decenal, terpineol, and citral were the VOCs that most strongly reduced the development of *Colletotrichum lindemuthianum*, *Fusarium oxysporum*, *Botrytis cinerea in vitro*, and methyl jasmonate, limonene and linalool had particularly strong inhibitory effects on the germination of *C. lindemuthianum* on the leaves of bean plants (Quintana-Rodriguez et al., 2015; Quintana-Rodriguez et al., 2018a). Therefore, no strict correlation of VOCs concentration in the wax matrix with the inhibitory effect was to be expected, at least not across different types of VOCs.

In summary, future work will be needed to obtain a more complete understanding of the role that VOCs sequestered by epicuticular waxes play in the overall resistance of a plant. More efforts will be required to determine the concentration of VOCs in the headspace of plants under natural conditions: information which would be crucial to determine the level of resistance that plants can gain from sequestered VOCs. Nevertheless, our results show that the exposure of wax-layers to VOCs can generate a transient inhibitory effect on germinating fungal spores that is independent of any putative resistance-related traits in a supporting plant. These antifungal effects of sequestered VOCs that act in the outermost layers of a leaf can represent a further mechanism *via* which exogenous VOCs contribute to the phenomenon of associational resistance. Therefore, our study serves as a proof of concept that supports a role of exogenous VOCs in the passive associational resistance of plants to pathogens. Our observations also indicate that stable concentrations of VOCs in the atmosphere might be less critical for their potential use in biological control than it has been assumed so far. In fact, our observation might not come as a too big surprise, at least not if we consider that waxes and lipids have historically been used to retain aromas: the sequestering of flower scents by waxes represents the basis of a classical technique in perfumery termed ‘*enfleurage*’ (Ribechini et al., 2008; Paibon et al., 2011) and served even as the central topic of Patrick Süsskind’s novel ‘The perfume’. Plants might commonly use ‘*enfleurage*’ for their own benefits. Therefore, future studies aimed at understanding associational resistance in plants should consider the potential effects of exogenous and endogenous VOCs that are sequestered by epicuticular waxes.

## Data Availability Statement

The raw data supporting the conclusions of this article will be made available by the authors upon request.

## Author Contributions

XC-C, JM-T, and MH conceived the ideas and designed different parts of methodology. XC-C performed the experiments and collected the data. XC-C and JM-T analysed the wax and VOC samples, and XC-C and MH wrote the manuscript. All authors contributed critically to the drafts and gave final approval for publication.

## Funding

This work was economically supported by CONACYT de México and by institutional support from CINVESTAV - Unidad Irapuato. The funders had no influence in the planning or execution of the research, the analysis of data or their presentation. MH and XC-C gratefully acknowledge financial support from CONACYT (grants 258119 to MH and 429106 to XC-C).

## Conflict of Interest

The authors declare that the research was conducted in the absence of any commercial or financial relationships that could be construed as a potential conflict of interest.
