# Supplementary material for: Sequestration of Exogenous Volatiles by Plant Cuticular Waxes as a Mechanism of Passive Associational Resistance: A Proof of Concept
Source: Front Plant Sci. 2020 Feb 25;11:121. doi: 10.3389/fpls.2020.00121 (PMC7052286; doi:10.3389/fpls.2020.00121)
Supplement: Supplementary file 1 [file DataSheet_1.docx]

**Mass spectra and preliminary identification of compounds in
chloroform-based extracts of the cuticular waxes of *Parkinsonia praecox***

**Figure A:** Mass spectra of the ten quantitatively dominant components of *Parkinsonia praecox* cuticular waxes that were preliminarily identified to the level of individual compound (peaks 2-11 in Figure 2A)

Peak 2. Tocopherol (triterpenoid 1). Retention time: 37.87, match 831

Peak 3. Nonacosane (alkane 1). Retention time: 39.81, match 923

Peak 4. Germanicol (triterpenoid 2). Retention time: 42.81, match 654

Peak 5. Triacontane (alkane 2). Retention time: 44.69, match 846

Peak 6. Hopenone b (triterpenoid 3). Retention time: 44.90, match 753

Peak 7. Lupenone (triterpenoid 4). Retention time: 46.23, match 931

Peak 8. Cyclolanostanol-derivative 1 (triterpenoid 5). Retention time: 48.47, match 682

Peak 9. Oxolanostadienoate-derivative (triterpenoid 6). Retention time: 49.09, match 676

Peak 10. Betulin (triterpenoid 7). Retention time: 49.92, match 695

Peak 11. Cyclolanolstanol-derivative 2 (triterpenoid 8). Retention time: 54.52, match 758

**Figure B:** Mass spectra of further, quantitatively minor components of *Parkinsonia praecox* cuticular waxes that were preliminarily identified to the level compound class (Figure 2C: 1 triterpenoid, 26 alkanes, 16 fatty acids, 4 alkenes and 3 alcohols).

Triterpenoid 9: Retention time: 33.50, match 566

Alkane 3: 5,7-Dimethyl-undecane. Retention time: 5.283, Match 864

Alkane 4: 2,3,6,7-Tetramethyl-octane. Retention time: 5.72, Match 883.

Alkane 5: 6-Ethyl-2-methyl-octane. Retention time: 5.78, Match 833

Alkane 6: 2,3,5,8-Tetramethyl-decane. Retention time: 6.9, Match 801

Alkane 7: 5-Ethyl-5-methyl-decane. Retention time: 7.04, match 867

Alkane 8: 4,6-Dimethyl-dodecane. Retention time: 7.35, Match 868

Alkane 9: 3,7-Dimethyl-undecane. Retention time: 7.53, match 754

Alkane 10: 2,6,11-Trimethyl-dodecane. Retention time: 7.71, match 721

Alkane 11: 2,7,10-Trimethyl-dodecane. Retention time: 7.87, Match 784

Alkane 12: 2,6,10-Trimethyl-dodecane. Retention time: 7.92, match 825

Alkane 13: 2,3,5,8-Tetramethyl-decane. Retention time: 8.68, match 803

Alkane 14: Tridecane. Retention time: 8.75, match 842

Alkane 15: 2-methyl-tridecane. Retention time: 8.82, match 799

Alkane 16: 4 methyl-tridecane. Retention time: 8.81, match 749

Alkane 17: Pentadecane. Retention time: 9.02, match 854

Alkane 18: Hexadecane. Retention time: 10.97, match 833

Alkane 19: 7-methyl-6-tridecene. Retention time: 11.16, match 800

Alkane 20: 2-methyl-heptadecane. Retention time: 11.45, match 775

Alkane 21: Nonadecane. Retention time: 13.82, match 822

Alkane 22: Eicosane. Retention time: 14.50, match 784

Alkane 23: Heneicosane. Retention time: 17.76, match 814

Alkane 24: Docosane. Retention time: 18.62, match 824

Alkane 25: 2,2,-Dimethyl-docosane. Retention time: 22.46, match 720

Alkane 26: Retention time: 30.70, match 857 (comm: molecule peak shows that annotation ‘hexacosane’ is incorrect)

Alkane 27: Hexacosane. Retention time: 32.95, match 860

Alkane 28: Octacosane, Retention time: 35.33, match 900

Fatty acid 1: Propanoic acid. Retention time: 5.341, match 921

Fatty acid 2: Benzoic acid. Retention time 7.11, match 899

Fatty acid 3: Octanoic acid. Retention time: 7.20, match 816

Fatty acid 4: Butanedioic acid. Retention time: 7.63, match 906

Fatty acid 5: Methyl-butanedioic acid. Retention time: 8.32, match 863

Fatty acid 6: Hexanedioic acid. Retention time: 9.09, match 788

Fatty acid 7: Ethyl 4-ethoxybenzoate. Retention time: 9.36, match 923

Fatty acid 8: Dodecanoic acid. Retention time: 10.34, match 930

Fatty acid 9: Tetradecanoic acid. Retention time: 12.65, match 803

Fatty acid 10, Retention time: 12.91, match 842

Fatty acid 11: Hexadecanoic acid. Retention time: 15.91, match 825

Fatty acid 12: 9,12(Z,Z)Octadecanoic acid. Retention time: 19.37, match 902

Fatty acid 13: 11-Trans-octadecanoic acid. Retention time: 19.48, match 905

Fatty acid 14: Octadecanoic acid, Retention time: 20.05, match 904

Fatty acid 15: Docosanoic acid. Retention time: 29.39, match 867

Fatty acid 16: Tetracosanoic acid. Retention time: 34.00, match 807

Alkene 1. Retention time: 11.74, match 800

Alkene 2: 3-Eicosene. Retention time: 11.90, match 908

Alkene 3: 5-Eicosene. Retention time: 14.92, match 928

Alkene 4: 1-Docosene. Retention time: 18.89, match 930

Alcohol 1: Glycerol. Retention time: 7.32, match 936

Alcohol 2: Retention time: 7.57, match 820

Alcohol 3: Xilitol. Retention time: 11.27, match 939
